# Randomized trial of intraoperative nitrous oxide for postoperative pain reduction in cervical spinal cord injury

**DOI:** 10.1097/PR9.0000000000001473

**Published:** 2026-08-05

**Authors:** Mayu Hoshina, Takao Kato, Yuki Shiko, Yohei Kawasaki, Kunihide Okubo, Akiko Kimura, Madoka Yokotani, Yusuke Mazda, Tadashi Yahata, Koichi Inokuchi, Hideaki Obata

**Affiliations:** a Department of Anesthesiology, Saitama Medical Center, Saitama Medical University, Kawagoe, Japan; b Department of Biostatistics, Graduate School of Medicine, Saitama Medical University, Moroyama, Japan; c Department of Obstetric Anesthesiology, Center for Maternal-Fetal and Neonatal Medicine, Saitama Medical Center, Saitama Medical University, Kawagoe, Japan; d Advanced Center for Emergency and Critical Care Medicine, Saitama Medical Center, Saitama Medical University, Kawagoe, Japan

**Keywords:** Nitrous oxide, Cervical spinal cord injury, Postoperative pain, Neuropathic pain, Central sensitization, Randomized clinical trial

## Abstract

**Introduction:**

Neuropathic pain after cervical spinal cord injury (SCI) is common and difficult to manage.

**Objectives:**

To evaluate whether intraoperative nitrous oxide (N_2_O) influences acute postoperative pain and long-term neuropathic pain after cervical SCI surgery.

**Methods:**

This single-center, randomized study enrolled adults (≥20 yr) undergoing emergency surgery for traumatic cervical SCI (American Spinal Injury Association Impairment Scale C or D). Patients were randomly assigned to receive intraoperative N_2_O or Air. The primary outcome was resting pain intensity on postoperative day 1 (D1), measured using the Numeric Rating Scale (NRS). Secondary outcomes included global pain and neuropathic pain assessments up to 6 months (6M) after surgery.

**Results:**

Of 80 randomized patients, 59 were analyzed (mean [SD] age 70.34 [12.07] years; 46 [78%] male). Baseline characteristics were similar between groups (Air, n = 29; N_2_O, n = 30). The primary outcome was higher in the N_2_O group, indicating no acute analgesic effect (NRS on D1: 3.27 [2.69] vs 5.10 [3.06]; *P* = 0.021). By contrast, at 6M, resting NRS was lower in the N_2_O group (1.72 [2.64] vs 0.53 [1.25]; *P* = 0.030), and numbness-related symptoms also improved. No serious adverse events occurred.

**Conclusion:**

Intraoperative N_2_O did not reduce acute postoperative pain after cervical SCI surgery. However, exploratory secondary analyses suggested a potential reduction in chronic neuropathic pain at 6 months, supporting the hypothesis that early perioperative modulation of central sensitization may influence pain chronification after SCI.

## Abstract 2

Supplemental Digital Content is Available in the Text.

Intraoperative nitrous oxide did not reduce acute postoperative pain but was associated with lower neuropathic symptoms at 6 months after cervical spinal cord injury surgery.

## 1. Introduction

Pain after cervical spinal cord injury (SCI) is notoriously difficult to treat.^5^ The nature of pain after cervical SCI varies widely and includes musculoskeletal, spastic, visceral, and neuropathic types; approximately 50% of patients experience neuropathic pain after cervical SCI.^5,12,21^ Surgical decompression improves neurological recovery, particularly when performed within 24 hours of injury, but does not reduce neuropathic pain at one year, as shown in a large multicenter cohort by Optimal Treatment for Spinal Cord Injury associated with Cervical Canal Stenosis (OSCIS) investigators.^16^ Causes of neuropathic pain include multiple factors such as N-methyl-D-aspartate (NMDA) receptor hyperactivation because of nerve damage, Na^+^ channel upregulation, dysfunction of gamma-aminobutyric acid-releasing neurons, and the descending inhibitory pathway.^2,13^

Nitrous oxide (N_2_O) is an inhalational anesthetic with antinociceptive properties mediated primarily through activation of the noradrenergic descending inhibitory pathway and noncompetitive antagonism of NMDA receptors in the spinal cord.^2,10,22,24^ NMDA receptors are significantly involved in central sensitization within the spinal cord and contribute to the development of chronic pain.^17^

Experimental and small clinical studies have suggested that N_2_O may modulate neuropathic pain; several-day inhalation has been reported to alleviate peripheral neuropathic allodynia with effects lasting up to 4 weeks.^4^ Furthermore, perioperative studies have indicated that intraoperative N_2_O may reduce the risk of chronic postoperative pain.^7^ However, its potential role in preventing chronic neuropathic pain after cervical SCI, a population at particularly high risk, has not been examined in a randomized trial.

Based on these findings, we hypothesized that early administration of nitrous oxide (N_2_O) during surgery for cervical SCI may reduce persistent nociceptive input, inhibit central sensitization, and prevent the transition from acute pain to chronic neuropathic pain. Our primary hypothesis was that intraoperative N_2_O administration would reduce resting pain intensity on postoperative day 1 compared with Air administration. Our secondary hypothesis was that intraoperative N_2_O would reduce the development of chronic neuropathic pain at 6 months. Additional secondary endpoints included neuropathic symptom profiles (Numeric Rating Scale for Neuropathic Pain [NRS-N], Neuropathic Pain Symptom Inventory [NPSI]), patient-reported global impression of change (PGIC), psychological status (Hospital Anxiety and Depression Scale [HADS]), and quality of life (EuroQol-5D-5L) during 6 months of follow-up.

This trial was designed to evaluate both acute and long-term pain outcomes, with secondary analyses intended to explore potential mechanisms underlying pain chronification after SCI.

## 2. Methods

### 2.1. Study design

This was a single-center, double-blind randomized controlled trial conducted at Saitama Medical Center, Saitama Medical University (Kawagoe, Japan) between May 2022 and December 2023. The study was approved by the Research Ethics Committee of Saitama Medical University General Medical Center (approval No. 2021-164-II) and prospectively registered in the Japan Registry of Clinical Trials (jRCT1031210580; https://jrct.mhlw.go.jp/en-latest-detail/jRCT1031210580) on January 25, 2022. The first patient was enrolled on May 11, 2022. All prespecified primary and secondary outcomes were consistent with the trial registration. Written informed consent was obtained from all participants.

### 2.2. Study population

Eighty adult patients undergoing urgent surgery for cervical SCI were enrolled.

Inclusion criteria were as follows: (1) Japanese-speaking adults ≥20 years old; (2) cervical SCI classified as ASIA Impairment Scale grade C or D; and (3) ability to understand the study and provide voluntary informed consent.

Exclusion criteria included: contraindications to N_2_O (eg, pneumothorax, head injury, history of blood disorders), concomitant extremity trauma, impaired consciousness, or active psychiatric disorders (eg, suicidal ideation).

### 2.3. Randomization and blinding

The random allocation sequence was generated by an anesthesiologist not involved in patient enrollment or outcome assessment using computer-generated block randomization (block size 4). Allocation was concealed using opaque, sealed envelopes prepared in advance. The attending anesthesiologist opened the envelope immediately before induction of anesthesia and was not involved in postoperative evaluations. Patients, outcome assessors, and investigators performing analyses were blinded to group assignment.

### 2.4. Study protocol

Pre-anesthetic assessment confirmed eligibility and obtained baseline resting pain using the Numerical Rating Scale (NRS; 0–10). No premedication was administered.

Standard monitoring (ECG, pulse oximetry, noninvasive blood pressure, and arterial pressure when indicated) was initiated on arrival to the operating room. After preoxygenation, anesthesia was induced with propofol, remifentanil, fentanyl, and rocuronium at the discretion of the attending anesthesiologist. Anesthesia was maintained with sevoflurane and continuous remifentanil infusion (0.1–0.25 μg/kg/min). Patients were positioned prone. The Air group received air–oxygen, whereas the N_2_O group received N_2_O–oxygen. In both groups, FiO_2_ was maintained at 0.4 (fresh gas flow 3 L/min). Hemodynamic management (ephedrine, phenylephrine, and norepinephrine) was performed as clinically indicated. At the end of surgery, intravenous acetaminophen (1000 mg) and ondansetron (4 mg) were administered. After emergence, patients received 100% oxygen, neuromuscular blockade was reversed with sugammadex (2–4 mg/kg), and patients were extubated and transferred to the intensive care unit. Pain and neuropathic symptoms were assessed at 4 time points: postoperative day 1 (1D), 2 weeks (2W), 3 months (3M), and 6 months (6M). All assessments were performed by physicians blinded to group assignment.

### 2.5. Outcomes

#### 2.5.1. Primary outcome

Resting pain intensity on postoperative day 1, measured using the Numerical Rating Scale (NRS).

#### 2.5.2. Secondary outcomes

Secondary outcomes included:

(1) Pain intensity and on movement (NRS-P) at 1D, 2W, 3M, and 6M
(2) Degree of numbness (NRS-N; 0–10)
(3) Neuropathic symptoms using the Neuropathic Pain Symptom Inventory (NPSI)
(4) Psychological status using the Hospital Anxiety and Depression Scale (HADS)
(5) Quality of life using the EQ-5D-5L
(6) Patient Global Impression of Change (PGIC)
(7) Relationship between time-to-surgery and postoperative pain

NRS-N, NPSI, and PGIC were prespecified as key secondary outcomes.

Outcomes 1–3 were assessed at 1D, 2W, 3M, and 6M; outcomes 4–6 at 2W, 3M, and 6M.

#### 2.5.3. Pain score definitions

(1) NRS-P at rest before surgery served as the baseline for resting pain.
(2) From 1D onward, pain at rest and pain on movement were evaluated separately.
(3) Changes from baseline were calculated as:
  ΔNRS = postoperative NRS − baseline NRS
(4) NRS-N and NPSI changes were calculated using 1D as the baseline.

#### 2.5.4. Assessment of adverse events

At 1D, sore throat, hoarseness, and postoperative nausea and vomiting (PONV) were recorded.

Hospital assessments were obtained in person, whereas 2W–6M evaluations were performed by telephone with standardized questionnaires.

### 2.6. Sample size

Based on a preliminary study, a sample size of 26 patients per group was required to detect a 2-point difference in the primary endpoint (SD 2.5), with 80% power and α = 0.05.^4^

Assuming a 30% loss to follow-up for long-term outcomes, the total sample size was set at 80 patients.

The intention-to-treat (ITT) population included all randomized patients with available POD1 pain data.

The modified intention-to-treat (mITT) population included all patients with available 6M outcomes.

### 2.7. Statistical methods

Parametric analyses were prespecified for the primary comparisons in this randomized controlled trial and were applied consistently regardless of the observed data distribution. Student t-test was used for between-group comparisons of continuous outcomes, and Pearson correlation coefficients were used to assess associations because these methods are generally robust to moderate deviations from normality in the absence of extreme skewness or outliers. To assess the robustness of the findings, additional nonparametric analyses (Wilcoxon rank sum test for between-group comparisons and Spearman rank correlation for associations) were also performed.

Continuous variables are presented as mean ± SD, and categorical variables as counts and percentages. All tests were 2-tailed, and *P* < 0.05 was considered statistically significant. Statistical analyses were performed using SAS version 9.4 (SAS Institute, Cary, NC). Primary outcome analyses (1D resting pain) were performed in the ITT population. Chronic outcomes (6 M neuropathic pain) were analyzed in the mITT population.

## 3. Results

**Figure 1.**
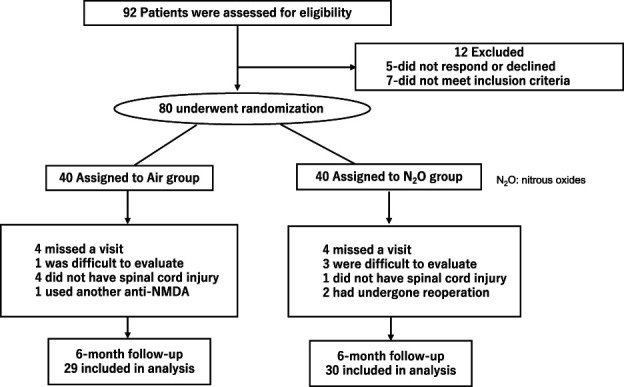
CONSORT flow diagram of patient enrollment, randomization, and follow-up.

The recruitment period was from May 2022 to May 2023, and the follow-up period was from May 2022 to December 2023, concluding with the completion of follow-up for the target number of participants (Fig. 1).

A total of 92 patients were screened; 12 were excluded (7 did not meet inclusion criteria and 5 declined participation). The remaining 80 patients were randomized to the Air group (n = 40) or the N_2_O group (n = 40).

Before the 6-month follow-up, 21 patients were excluded:

(1) lost to follow-up: 8 (Air/N_2_O: 4/4)
(2) cognitive decline: 4 (Air/N_2_O: 1/3)
(3) reoperation: 2 (1/1)
(4) additional intraoperative NMDA antagonist: 2 (1/1)

Primary outcome analyses were conducted in the intention-to-treat population, whereas 6-month outcomes were analyzed in the modified intention-to-treat population (n = 59). The mean (SD) age was 70.3 (12.1) years, and approximately two-thirds of participants were male. Baseline characteristics, including comorbidities and injury etiology, were comparable between groups (Table 1). Falls were the most common cause of injury, consistent with national epidemiologic data on traumatic cervical spinal cord injury in Japan.^19^ Intraoperative opioid use, time from injury to surgery, and postoperative analgesic administration did not differ significantly between the 2 groups.

**Table 1:** Clinical characteristics of the patients and perioperative variables.

	Air (n = 29)	N_2_O (n = 30)
Age (y)	71.66 ± 13.15	69.07 ± 10.88
Sex (male/female)	23/6	23/7
ASIA class (C/D)	6/22	5/26
BMI (kg/m^2^)	23.37 ± 3.76	23.92 ± 3.62
Duration of starting surgery (h)	21.52 ± 13.03	26.12 ± 26.87
Anesthetics during surgery		
Remifentanil (mg)	1.31 ± 0.5	1.13 ± 0.5
Fentanyl (µg)	322.41 ± 125.06	331.67 ± 128.98
Ephedrine (mg)	17.52 ± 11.63	20.20 ± 11.10
Phenylephrine (mg)	0.34 ± 0.54	0.47 ± 0.45
Norepinephrine (mg)	0.17 ± 0.25	0.22 ± 0.3
Nitrous oxide (L)	—	542.83 ± 555.05
Pre-existing cervical spine disease		
None	17	21
OPLL	9	6
Cervical spondylosis	3	3
Cause of injury		
Fall	21	17
Fall down	5	6
Traffic accident	3	7
Postoperative analgesics (during hospitalization)		
NSAIDs	9	13
Acetaminophen	4	2
Pregabalin/mirogabalin	5	10
Tramadol	15	18
Fentanyl	10	6

Baseline characteristics were generally comparable between patients who completed follow-up and those lost to follow-up. Although a difference was observed in preexisting cervical spine conditions, this may reflect a chance imbalance given the small sample size. These detailed comparisons are presented in e-Table 4 (supplemental digital content, http://links.lww.com/PR9/A424).

## 4. Pain intensity

Preoperative resting NRS-P did not differ between groups (Air: 6.2 vs N_2_O: 6.2).

### 4.1. Acute pain

**Figure 2.**
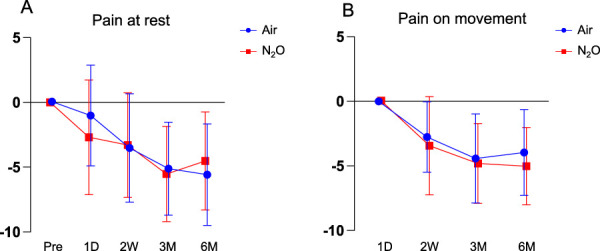
Change in postoperative pain intensity measured with the Numerical Rating Scale (NRS) over 6 months in the Air (blue) and N_2_O (red) groups. (A) Resting NRS scores from preoperative baseline (Pre) to postoperative day 1 (1D), 2 weeks (2W), 3 months (3M), and 6 months (6M). (B) NRS scores on movement from baseline at postoperative day 1 (1D; 24–36 hours after surgery) to 2W, 3M, and 6M. Data are presented as mean ± SD.

On postoperative day 1, resting NRS-P was significantly higher in the N_2_O group (mean difference [MD] 1.8; 95% CI, −3.4 to −0.3; *P* = 0.021), indicating that intraoperative N_2_O did not confer an acute analgesic effect (Fig. 2A; e-Table 1, supplemental digital content, http://links.lww.com/PR9/A424).

### 4.2. Chronic pain

At 6 months, resting NRS-P was significantly lower in the N_2_O group (MD 1.2; 95% CI, 0.1–2.3; *P* = 0.030).

There were no significant differences at 2 weeks or 3 months (Fig. 2A).

Pain on movement did not differ significantly at any time point (Fig. 2B).

### 4.3. Neuropathic pain assessment

Neuropathic pain assessed by NRS-N and NPSI showed no group differences on day 1.

**Figure 3.**
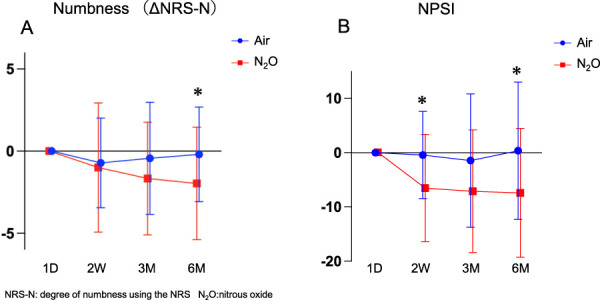
Change in neuropathic symptoms over 6 months in the Air (blue) and N_2_O (red) groups. (A) Change in numbness intensity (ΔNRS-N) from postoperative day 1 (1D) baseline to 2W, 3M, and 6M. (B) Change in Neuropathic Pain Symptom Inventory (NPSI) total scores from 1D baseline to 2W, 3M, and 6M. Data are presented as mean ± SD. *P* < 0.05 was considered statistically significant.

At 6 months, however:

(1) NRS-N was significantly lower in the N_2_O group (MD 1.8; 95% CI, 0.5–3.2; *P* = 0.010; Fig. 3A)
(2) NPSI total scores improved at 2 weeks and at 6 months (Fig. 3B; e-Table 1, supplemental digital content, http://links.lww.com/PR9/A424)
(3) However, the between-group difference in NRS-N was not statistically significant in nonparametric analysis (Wilcoxon rank sum test, *P* = 0.0608).

Subcomponent analysis revealed:

(1) Higher evoked pain in the N_2_O group on day 1
(2) Lower abnormal sensations at 6 months (e-Table 2, supplemental digital content, http://links.lww.com/PR9/A424)
  Abnormal sensations remained prevalent throughout follow-up (76%–90%, e-Table 3, supplemental digital content, http://links.lww.com/PR9/A424).

### 4.4. Other outcomes

#### 4.4.1. Patient-reported global impression of change

Patient-reported global impression of change scores at 6 months indicated better perceived improvement in the N_2_O group (mean 1.72 vs 2.30; *P* = 0.033) (Table 2).

**Table 2:** Change from baseline of Hospital Anxiety and Depression Scale (HADS), EuroQOL 5 dimensions-5L (EQ-5D-5L), and Patient Global Impression of Change (PGIC) scores in the N_2_O and Air groups over the 6-mo follow-up.

	Air (n = 29)	N_2_O (n = 30)	*P*
HADS anxiety			
2W	4.74 (3.43)	2.93 (2.92)	**0.047** *
3M	1.88 (3.02)	1.50 (1.62)	0.559
6M	1.88 (1.83)	2.14 (2.66)	0.684
HADS depression			
2W	9.00 (4.83)	6.00 (5.24)	**0.040** *
3M	5.15 (4.01)	3.75 (3.75)	0.189
6M	3.60 (3.18)	3.41 (3.27)	0.833
EQ5D-5L			
2W	0.38 (0.29)	0.48 (0.27)	0.222
3M	0.57 (0.25)	0.66 (0.21)	0.167
6M	0.54 (0.26)	0.66 (0.20)	0.073
PGIC			
2W	1.96 (0.64)	1.67 (1.09)	0.274
3M	1.81 (0.85)	1.68 (1.19)	0.650
6M	2.30 (1.10)	1.72 (0.84)	**0.033** *

Data are presented as mean and SD.

* *P* < 0.05 from the Air group.

Nonparametric analysis (Wilcoxon rank sum test) did not show statistically significant between-group differences for HADS scores.

The significance of the bold entries is indicated by the table footnote (*P<0.05 from the air group).

#### 4.4.2. HADS, EQ-5D-5L

Exploratory analyses of HADS and EQ-5D-5L revealed inconsistent or nonsignificant between-group differences.

#### 4.4.3. Time to surgery

No meaningful correlation was observed between time-to-surgery and pain intensity at any time point.

### 4.5. Adverse events

No significant difference was found between the groups in the incidence of PONV, sore throat, or hoarseness. The incidence of PONV during the perioperative period is reported to be around 30%,^11^ and in the present study, it was 29.9% in the Air group and 23.4% in the N_2_O group, consistent with previous reports.

## 5. Discussion

Neuropathic pain after cervical spinal cord injury (SCI) is a major contributor to long-term disability and reduced quality of life, yet effective preventive strategies remain limited. This exploratory randomized trial investigated whether intraoperative nitrous oxide (N_2_O), a minimally invasive anesthetic adjunct with N-methyl-d-aspartate (NMDA) receptor–blocking properties, influences acute postoperative pain or long-term neuropathic pain trajectories.

In this study, the prespecified primary outcome—resting pain on postoperative day 1—was higher in the N_2_O group, indicating that N_2_O does not attenuate acute postoperative pain after cervical SCI surgery. Acute postoperative pain is largely determined by tissue injury, inflammation, and perioperative opioid exposure rather than NMDA receptor–mediated pathways. Therefore, the absence of an acute analgesic effect is biologically plausible and consistent with previous investigations of N_2_O in acute postoperative pain.^8,9,15,20^ Small imbalances in injury severity (a greater proportion of high-energy trauma in the N_2_O group) and differences in perioperative opioid administration may also have contributed to this finding.

By contrast, secondary outcomes suggested a potential long-term benefit of N_2_O on neuropathic pain. At 6 months, patients who received N_2_O reported lower numbness and abnormal sensation scores, and PGIC showed a parallel trend. Although this study was not powered to detect long-term differences, the observed improvements represent an exploratory mechanistic signal consistent with modulation of central sensitization. NMDA receptors play a crucial role in the transition from acute to chronic pain,^18^ and previous animal studies have demonstrated that brief N_2_O exposure can exert sustained antihyperalgesic effects in neuropathic pain models^1,3^

This dissociation between acute postoperative pain and later neuropathic symptoms supports the concept that early modulation of spinal excitability may influence pain chronification independently of immediate nociceptive pain intensity.

Further mechanistic studies examining spinal cord excitability and central sensitization before and after intervention would help clarify the biological basis of the observed long-term effects.

Clinical evidence regarding N_2_O and chronic pain has been mixed: some studies have shown reductions in allodynia,^4^ whereas others—including those in complex regional pain syndrome—have not.^14^ However, several perioperative studies, including ENIGMA II, have suggested that intraoperative N_2_O may reduce the risk of chronic postoperative pain in certain populations.^6,23^ The present findings extend these observations to a high-risk group with substantial susceptibility to neuropathic pain.

Because surgically treated traumatic cervical SCI is rare, recruitment was inevitably limited, and the modest sample size reflects the practical constraints of enrolling this population rather than a weakness in study design. Importantly, primary endpoint data were available for nearly all participants, which supports the internal validity of the acute pain findings. The exploratory signal in neuropathic pain warrants attention, as early alterations in spinal cord excitability may contribute to later chronic pain development.^22^ Early N_2_O administration—typically within hours after injury—may influence such early pathophysiological processes.

Genetic factors may also modify the effect of N_2_O. Previous studies have suggested enhanced chronic pain reduction among Asian patients exposed to intraoperative N_2_O^6^, possibly related to the methylenetetrahydrofolate reductase (MTHFR) 677C>T polymorphism, which affects folate metabolism and may interact with the biochemical effects of N_2_O. Future studies incorporating genetic profiling are needed.

## 6. Limitations

This study has several limitations. First, it was conducted at a single center, and the sample size—particularly for long-term follow-up—was modest, which may limit the generalizability of the findings. Second, chronic-phase assessments were based on self-reported questionnaires and telephone interviews rather than in-person neurologic examinations, which may introduce measurement variability. Third, perioperative and postoperative analgesic management was not fully standardized, and incomplete documentation of postoperative interventions—particularly post-discharge medication use—limited our ability to adjust for potential confounders. Although randomization reduces the risk of confounding, residual imbalances in factors such as injury severity and postoperative analgesic use may still have influenced the results. In addition, the relatively small sample size further limited the feasibility and reliability of fully adjusted multivariable analyses. Accordingly, we did not perform such analyses in this study, and the findings should be interpreted with caution. The results should be interpreted as hypothesis-generating rather than confirmatory.

Future studies could address these limitations by conducting multicenter randomized controlled trials with larger cohorts to improve statistical power and generalizability. In addition, incorporating objective quantitative assessments, such as quantitative sensory testing or neuroimaging, would further strengthen the evaluation of neuropathic pain mechanisms.

## 7. Conclusion

Intraoperative nitrous oxide inhalation during cervical SCI surgery did not reduce acute postoperative pain. However, it was associated with lower neuropathic pain scores—particularly numbness and abnormal sensations—at 6 months, as well as greater patient-reported global improvement. Although these findings are exploratory, they suggest that early exposure to N_2_O may attenuate the development of chronic neuropathic pain in patients at high risk after cervical SCI.

Although the clinical use of N_2_O has declined in some regions because of environmental concerns, NMDA receptor antagonism remains relevant to the mechanistic understanding of postoperative neuropathic pain. Further studies with larger, multicenter cohorts are warranted to confirm these observations and clarify the underlying mechanisms.

## Disclosures

The authors have no conflict of interest to declare.

## Supplemental digital content

Supplemental digital content associated with this article can be found online at http://links.lww.com/PR9/A424.
